# Lamin A/C Mutants Disturb Sumo1 Localization and Sumoylation *in Vitro* and *in Vivo*

**DOI:** 10.1371/journal.pone.0045918

**Published:** 2012-09-21

**Authors:** Émilie Boudreau, Sarah Labib, Anne T. Bertrand, Valérie Decostre, Pierrette M. Bolongo, Nicolas Sylvius, Gisèle Bonne, Frédérique Tesson

**Affiliations:** 1 Interdisciplinary School of Health Sciences, University of Ottawa, Ottawa, Ontario, Canada; 2 UMRS 974, Inserm, Paris, France; 3 Université Pierre et Marie Curie-Paris Institut de Myologie, Paris, France; 4 Service de Biochimie Métabolique, U.F. Cardiogénétique et Myogénétique, AP-HP Groupe Hospitalier Pitié-Salpêtrière, Paris, France; “Mario Negri” Institute for Pharmacological Research, Italy

## Abstract

A-type lamins A and C are nuclear intermediate filament proteins in which mutations have been implicated in multiple disease phenotypes commonly known as laminopathies. A few studies have implicated sumoylation in the regulation of A-type lamins. Sumoylation is a post-translational protein modification that regulates a wide range of cellular processes through the attachment of small ubiquitin-related modifier (sumo) to various substrates. Here we showed that laminopathy mutants result in the mislocalization of sumo1 both *in vitro* (C2C12 cells overexpressing mutant lamins A and C) and *in vivo* (primary myoblasts and myopathic muscle tissue from the *Lmna^H222P^* ^/*H222P*^ mouse model). In C2C12 cells, we showed that the trapping of sumo1 in p.Asp192Gly, p.Gln353Lys, and p.Arg386Lys aggregates of lamin A/C correlated with an increased steady-state level of sumoylation. However, lamin A and C did not appear to be modified by sumo1. Our results suggest that mutant lamin A/C alters the dynamics of sumo1 and thus misregulation of sumoylation may be contributing to disease progression in laminopathies.

## Introduction

Mutations in the lamin A/C gene (*LMNA*) are associated with a collection of over ten diseases known as laminopathies, including but not restricted to dilated cardiomyopathy (DCM), Emery-Dreifuss muscular dystrophy (EDMD), restrictive dermopathy, familial partial lipodystrophy (FPLD), and premature ageing (OMIM # 150330). No definitive genotype-phenotype relationship exists for the over 400 mutations reported in this gene (for the full list of mutations, see UMD-*LMNA* database at www.umd.be/LMNA/). *LMNA* is one of the most frequently reported mutated genes in familial DCM and is associated with a worse prognosis than other forms of DCM [1]. Furthermore, mutations in *LMNA* are often responsible for an earlier onset and more severe form of EDMD compared to X-linked EDMD [2].

*LMNA* encodes the A-type lamin proteins, lamins A and C, which are expressed in all terminally differentiated and nucleated cells. Lamins A and C are type V intermediate filament proteins that constitute major components of the inner nuclear lamina. They play essential structural roles, such as nuclear membrane strength and shape, positioning of the nuclear pore complexes (NPCs), anchoring chromatin, and lamina assembly [3], [4], [5]. They also support regulatory roles as they are required for proper DNA transcription and gene expression [6], [7].

Investigations into the molecular consequences of *LMNA* mutations have shown that point mutations can affect lamina, filament, and protein complex assembly in a mutation-dependent manner [8], [9], [10], [11]. Expression of certain lamin A/C mutants results in intranuclear aggregations of the lamins while others do not appear to affect assembly or localization of the protein [12], [13]. Furthermore, mutations in *LMNA* can result in different cellular phenotype depending on which lamin A or C bears the mutation [10]. The absence of the lamin A/C protein causes the mislocalization of other nuclear proteins, such as emerin [14].

Our previous work demonstrated that *in vitro* over-expression of the lamin C isoform containing the p.Asp192Gly DCM-linked mutation resulted in the mislocalization and trapping of a post-translational protein modifier known as Small Ubiquitin-related MOdifier-1 (sumo1) inside lamin C aggregates [12]. The sequestration of sumo1 was abolished by the disruption of the sumo1 di-glycine motif required for sumoylation [12]. Sumoylation, which involves the covalent but reversible attachment of the ∼10 kD sumo1 protein to a lysine residue on the target protein, has been shown to regulate an assortment of cellular processes that include transcriptional regulation and nucleocytoplasmic transport [15], [16].The ubiquitin-conjugating enzyme E2I (Ubc9), which conjugates sumo1 to target proteins, interacts with lamin A/C [17]. Taken together these findings suggest that lamin A/C may be modified by sumo1 and that mutations in *LMNA* may disturb the sumoylation process by disrupting sumo1 localization. Interestingly, in HeLa cells and mouse cardiomyocytes, lamin A/C is preferentially modified by sumo2/3 over sumo1 and a DCM-associated mutation decreased the sumoylation [18]. Although grouped as members of the same family, while sumo2 and sumo3 show 97% sequence identity, sumo2/3 and sumo1 share only approximately 50% of their sequence and have distinct target sets, indicative of their distinct functions [19].

In the present study, we explore the role sumoylation plays in the pathogenesis of striated muscle related laminopathies. We tested the sumoylation of lamin A/C by sumo1 and investigated whether the expression of DCM- and EDMD- associated mutant lamin A and C alter the localization of sumo1 and disturb the sumo1 sumoylation process in the C2C12 mouse myoblast cell model. We confirmed our results using primary myoblasts isolated from the *Lmna* ^H222P/H222P^ knock-in mouse, which develops adult-onset muscle dystrophy and DCM comparable to the human phenotype [20], and validated the sumo1 mislocalization results *in vivo* in soleus muscle tissue biopsies from the *Lmna* ^H222P/H222P^ mouse. Our results further unveil the involvement of the sumo1 sumoylation pathway in the pathophysiology of laminopathies, especially those involving striated muscles.

## Results

To investigate the impact of *LMNA* mutations on the sumo1 sumoylation pathway, we examined sumoylation of lamin A/C and sumo1 localization in the presence of wild-type lamin A and C as well as DCM-associated p.Leu85Arg, p.Asp192Gly, and p.Gln353Lys lamin A and C mutants and EDMD-associated p.Arg386Lys and p.His222Pro lamin A and C mutants.

### Lamin A and C are not Sumoylated by Sumo1

**Figure 1:**
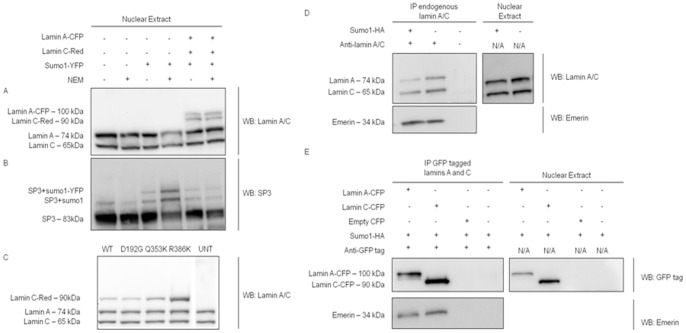
Lamin A and C are not sumoylated by sumo1. (A–C) Western blots of C2C12 nuclear proteins: (A) Untransfected (UNT) or sumo1-YFP transfected, or triple transfected wild-type lamin A-CFP, DsRed2-lamin C and sumo1-YFP probed for lamin A/C. NEM was included with harvesting of selected samples to stabilize sumo conjugation. (B) Western blot A stripped and reprobed for the reversibly sumoylated protein, SP3. (C) UNT or triple transfected wild-type and mutant DsRed2-lamin C, wild-type sumo1-YFP and wild-type Ubc9-HA probed for lamin A/C. (D–E) Immunoprecipitation of endogenous and exogenous lamin A and C. (D) Nuclear extracts of sumo1-HA transfected C2C12 cells were immunoprecipitated using anti-lamin A/C antibody and western blotting was performed for lamin A/C. (E) Nuclear extracts of lamin A-CFP, lamin C-CFP, or empty CFP vector transfected COS7 cells were immunoprecipitated using anti-GFP tag antibody and western blotting was performed for the GFP tags. All protein was harvested in the presence of NEM. Emerin, a known lamin A/C binding partner, was included as a positive control for immunoprecipitation.

We previously reported the disruption of sumo1 localization in COS7 cells over-expressing p.Asp192Gly mutant lamin C isoform and demonstrated that this mislocalization was sumoylation dependent [12]. Using SUMOsp 2.0.4 algorithm [21] to predict sumoylation sites, we identified two potential sumoylation consensus sites (type I: Ψ-K-X-E) at lysine amino acid positions 171 and 201, as well as three others (type II: non-consensus) at position 32, 260, and 420. Type I consensus sites are found more frequently modified by sumo1 and consist of a four residue motif, Ψ-K-X-E/D, where Ψ is any large hydrophobic amino acid, K is the target lysine to which sumo1 is attached, X is any amino acid, and E/D is aspartic or glutamic acid. To assess whether lamin A and C were sumoylated, we over-expressed tagged lamin A and lamin C in C2C12 cells. Due to the transient nature of sumoylation and the low steady-state levels of sumoylated proteins [22], cells were also transfected with tagged sumo1. However, no slower migrating bands corresponding to sumoylated lamin A or lamin C by either endogenous or exogenous sumo1 were observed (Figure 1A). To ensure the result was not cell-type specific, we confirmed the results using COS7 cells (data not shown). As a positive control of our protocol and of the sumoylation process in the C2C12 cell line, the blot was stripped and re-probed for the reversibly sumoylated protein, SP3 [23], [24]. We observed bands for SP3 sumoylated by both endogenously and exogenously expressed sumo1 (Figure 1B).

Given that we previously demonstrated how p.Asp192Gly mutant lamin C sequesters sumo1 inside aggregates, we investigated whether the expression of this mutant and other disease-associated mutant lamin C result in lamin’s sumoylation. We transfected tagged proteins: lamin C, sumo1, and Ubc9. Ubc9, the sumoylation conjugating enzyme, was co-transfected to further promote protein sumoylation. No bands corresponding to sumoylated lamin C were observed with any of the lamin C wild-type or mutants (Figure 1C), suggesting that wild-type as well as the other lamin A and C mutants are not sumoylated. To confirm this result, co-immunopreciptations were performed. In C2C12 cells transfected with tagged sumo1, immunoprecipitation of endogenous lamin A/C did not reveal lamin-sumo1-conjugates (Figure 1D). These results were confirmed in COS7 cells transfected with tagged wild-type lamin A, lamin C and with sumo1 (Figure 1E). Over-expression of ubc9 did not produce different results (data not shown).

### Mutant Lamin A/C Expression in C2C12 Cells Alters Localization of Sumo1 and ubc9

**Figure 2:**
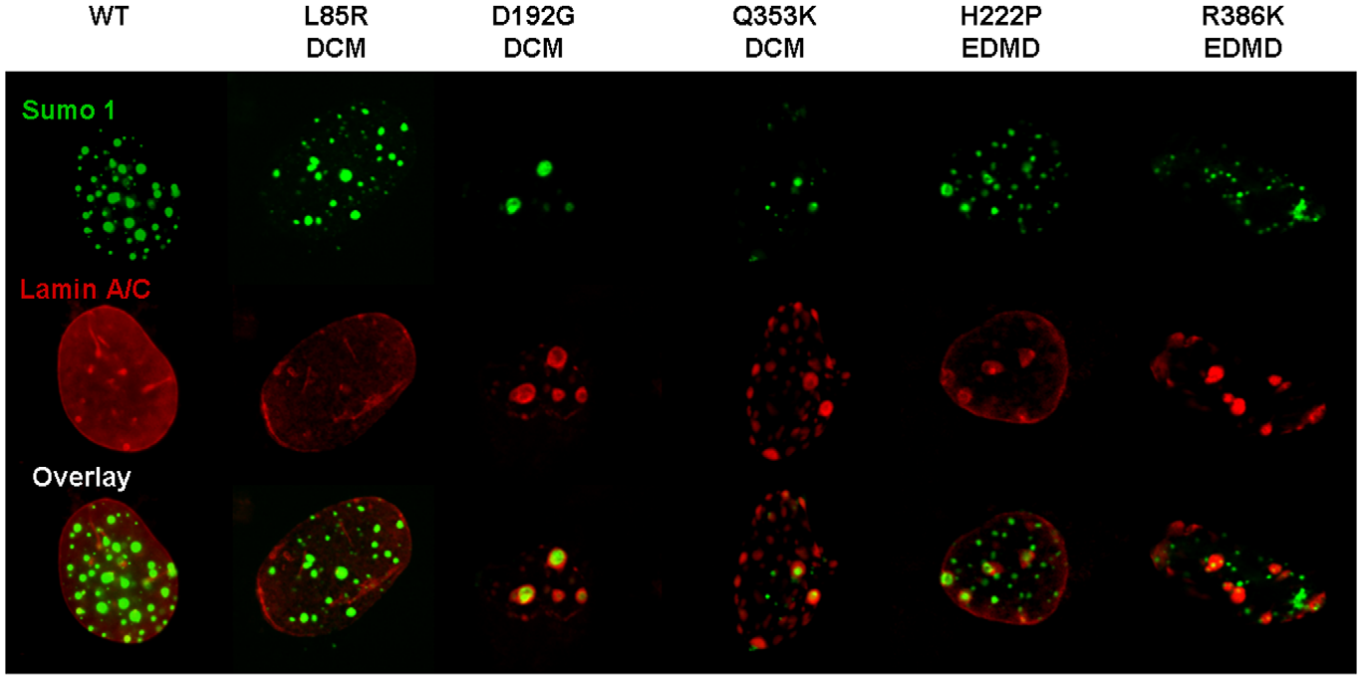
Sumo1 localization is disturbed by mutant lamin A/C in a mutation-dependent manner. Confocal microscopy images of nuclei of C2C12 cells expressing wild-type and mutant lamin A-CFP, DsRed2-lamin C and wild-type sumo1-YFP. The lamin constructs used as well as the associated pathology are indicated at the top. Lamin A and lamin C images are represented in one panel as they co-localize in all cells. Cells were visualized by wide-field fluorescence microscopy with excitation wavelengths of 434 nm for lamin A-CFP, 558 nm for DsRed2-lamin C, and 514 nm for wild-type sumo1-YFP. Each picture presented is representative of the most commonly observed phenotype.

**Figure 3:**
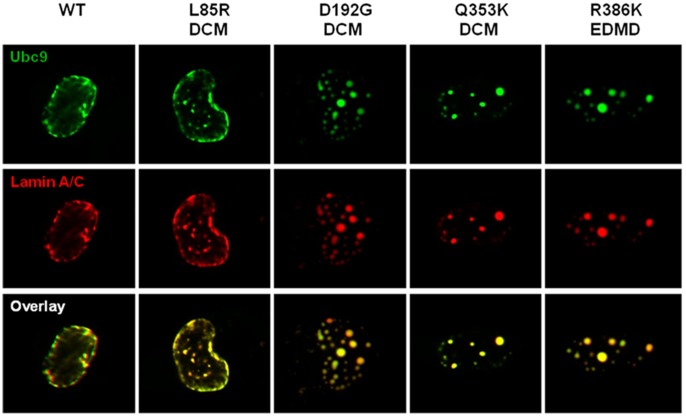
Ubc9 co-localizes with wild-type and mutant lamin A/C. Confocal microscopy images of nuclei of C2C12 cells expressing wild-type and mutant lamin A-CFP, DsRed2-lamin C and ubc9-GFP. The lamin constructs used as well as the associated pathology are indicated at the top. Lamin A and lamin C images are represented in one panel as they co-localize in all cells. Cells were visualized by wide-field fluorescence microscopy with excitation wavelengths of 434 nm for lamin A-CFP, 558 nm for DsRed2-lamin C, and 588 nm for ubc9-GFP. Each picture presented is representative of the most commonly observed phenotype.

We next investigated the effect of disease-associated *LMNA* point mutations on the localization of sumo1 and ubc9 in C2C12 cells. Wild-type lamin A, lamin C, and sumo1 show an even distribution in the nucleus (Figure 2). Since stoichiometry between lamin A and C has been shown to influence lamin A and C distribution within the nucleus [10], in order to maintain the stoichiometry of lamin A and C, we co-transfected lamin A and lamin C. In agreement with previously published results in other cell lines, the p.Leu85Arg mutant lamin A/C shows a comparable phenotype to the wild-type samples, and the p.Asp192Gly mutant lamin A/C results in nuclear aggregation of co-localized lamin A and C (Figure 2, Table 1). As shown previously with only p.Asp192Gly lamin C [12], expression of p.Asp192Gly lamin A and C aggregates disturb the localization of sumo1 by sequestering it within the aggregates (Figure 2, Table 1). The p.Gln353Lys substitution results in variable sizes and distributions of aggregated lamins A and C within the nucleus as well as at the nuclear periphery. This mutant also sequesters sumo1 within some of the aggregates (Figure 2, Table 1). As previously reported, the p.Arg386Lys mutation results in the formation of lamin A/C aggregates and we demonstrate here the trapping of the sumo1 protein (Figure 2, Table 1). The p.His222Pro mutant lamin A/C retains the ability to localize to the nuclear lamina but also develops aggregates with sumo1 sequestration (Figure 2). Ubc9 (either endogenous or transiently co-transfected ubc9-GFP) was observed to always co-localize with both wild-type and any mutant lamin A/C (Figure 3).

**Table 1:** Quantitative analysis of colocalization of sumo1 and mutant lamin A and C.

	D192G	Q353K	H222P	R386K
**tM1**	0.5057	0.4727	0.3632	0.7560
**tM2**	0.2500	0.2405	0.2889	0.6311
**% Ch1 int > thresh**	93.29	88.66	44.20	79.79
**% Ch2 int > thresh**	88.20	77.39	45.27	73.93

Quantitative colocalization analysis of sumo1 and lamin A and C was performed using the ImageJ- 1.46r software. Thresholded Manders coefficients tM1 and tM2 and the percentage of pixels intensity above threshold colocalized (% Ch1 or Ch2 int > thresh) in each of the two channels (Ch1 for sumo (green fluorescence) and Ch2 for lamin A and C (red fluorescence) were calculated using the threshold algorithm of Costes et al (2004) [43].

### Mutant Lamin A/C Expression Alters Cellular Sumoylation in C2C12 Cells

**Figure 4:**
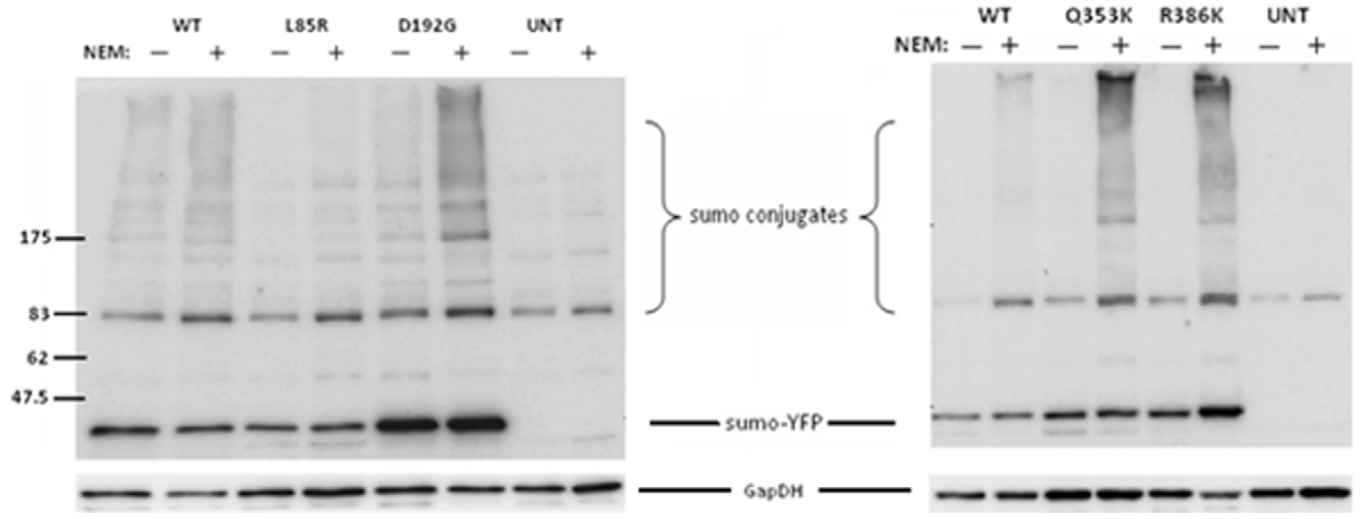
Mutant lamin A/C expression increases levels of sumoylated proteins. Western blot analysis of sumo1 in nuclear protein harvested from C2C12 untransfected (UNT) cells or transfected with wild-type (WT) or mutant lamin C-CFP and sumo1-YFP harvested with NEM. Blots were re-probed for GapDH as a loading control.

Given that mutant lamin A/C affected sumo1 localization, we next investigated the effect of p.Leu85Arg, p.Asp192Gly, p.Arg353Lys, and p.Arg386Lys lamin A/C mutations on the steady-state level of sumoylation. Western blotting was performed on nuclear extracts from C2C12 cells transfected with tagged lamin A, lamin C and sumo1 (n = 6) or lamin C, ubc9 and sumo1 (n = 2). Cells expressing mutant lamin A/C that trapped sumo1 in aggregates also showed an increase in steady-state sumoylation levels and non-conjugated sumo1-YFP (Figure 4). Densitometry analysis was performed on the bands corresponding to proteins conjugated to sumo1 (>85 kDa) and also on the band of non-conjugated sumo1 tagged with YFP (at ∼41 kDa). There was a statistically significant (P<0.002) increase in the steady-state levels of conjugated sumo1-YFP in the cells expressing p.Asp192Gly, p.Gln353Lys, and p.Arg386Lys mutant lamin A/C as compared to cells expressing wild-type lamin A and C. There was also an increase in the levels of non-conjugated sumo1-YFP in the cells expressing the p.Arg386Lys mutant lamin A/C (P<0.002) though the trend was less pronounced and fell slightly below statistical significance for the p.Asp192Gly (and p.Gln353Lys mutant lamin A/C (P<0.025).

### Sumo1 Localization and Sumoylation are Perturbed in *Lmna* ^H222P/H222P^ Mouse Myoblasts

**Figure 5:**
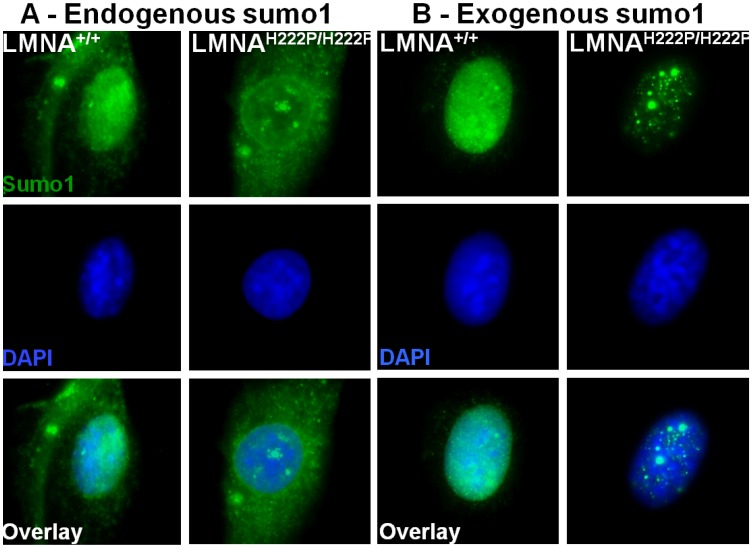
Sumo1 localization is disturbed by endogenous p.*His222Pro* mutant lamin A/C in primary mouse myoblasts. Fluorescent microscopy images of non-transfected and sumo1-YFP transfected *Lmna* ^+/+^ (WT) and *Lmna* ^H222P/H222P^ primary myoblast nuclei. (A) Untransfected myoblasts immunostained for endogenous sumo-1 (green). (B) Sumo1-YFP transfected myoblasts expressing YFP-tagged sumo1 (green). All myoblasts were counterstained for DAPI (blue). Each picture presented is representative of the most commonly observed phenotype.

In order to confirm our results in a physiologically relevant model, we cultured primary skeletal muscle myoblasts harvested from *Lmna* ^+/+^ and *Lmna* ^H222P/H222P^ mice and stained for sumo1 (Figure 5A). In *Lmna* ^+/+^ myoblasts, sumo1 shows homogenous nuclear and cytoplasmic distribution (Figure 5A). Approximately 75% of myoblasts of *Lmna* ^H222P/H222P^ mice show sumo1 localizing into nuclear foci (Figure 5A). Mutated myoblasts transfected with tagged sumo1 demonstrated an exacerbation of the foci phenotype to approximately 87% (Figure 5B).

### Altered Localization of Endogenous Sumo1 in *Lmna* ^H222P/H222P^ Mouse Muscle Tissue

**Figure 6:**
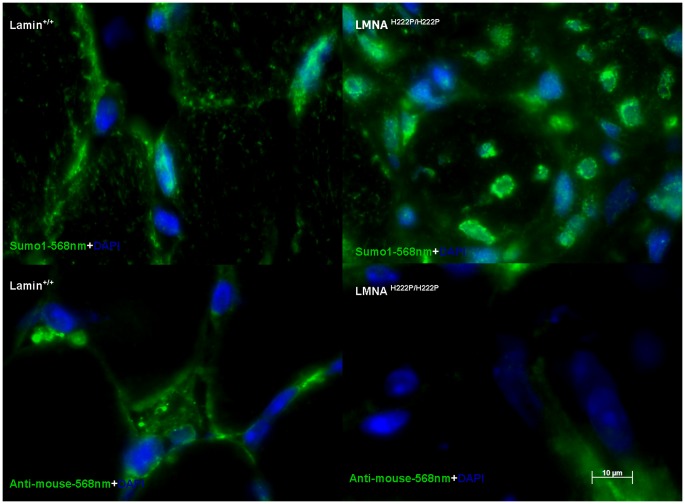
Sumo1 localization is disrupted in soleus muscle sections from *Lmna* ^H222P^ mice. Fluorescent microscopy images of soleus muscle cross sections from *Lmna* ^+/+^ and *Lmna* ^H222P/H222P^ mice. Top row sections were stained for sumo1 (green). Bottom row sections were stained with only secondary antibody to show background signal of anti-mouse-568 nm antibody (green). All sections were counterstained for DAPI (blue). Each picture presented is representative of the most commonly observed phenotype. Scale bar represents 10 µm. White arrow highlights one cell with nuclear aggregation of sumo1.

As endogenous sumo1 was found mislocalized in myoblasts cultured from *Lmna* ^H222P/H222P^ mice, we wanted to determine if this occurred *in vivo* in dystrophic *Lmna* ^H222P/H222P^ muscle tissue [20]. Therefore we examined its localization directly by immunostaining for sumo1 in cross-sections of soleus muscle from *Lmna* ^+/+^ and *Lmna* ^H222P/H222P^ mice (Figure 6). Wild-type muscle tissue sumo1 shows consistent punctate myocyte staining and homogenous punctate nuclear localization (Figure 6 left panel) in 87% of nuclei that is comparable to endogenous myoblast sumo1 staining (Figure 5A). In the p.His222Pro mutant muscles, we observed a non homogeneous localization of sumo1 including intranuclear and nuclear envelope aggregates in 43% of nuclei (Figure 6 right panel). The cytoplasmic localization of sumo1 appears conserved in most cells.

## Discussion

The lamin A/C protein interacts with the conjugating enzyme, ubc9 [17] and its sequence contains five putative sumoylation sites, yet we could not detect its sumoylation by sumo1. This is in agreement with the study that found lamin A/C to be preferentially modified by sumo2/3 [18]. The mutations included in this study are located in the α-helical coiled coil rod domains of lamin A/C that are essential for both filament assembly and protein-protein interactions [25]. As we previously demonstrated the aggregation of lamin C and the sequestration of sumo1 [12], we investigated whether mutations in lamin C would produce a misfolding of the protein that might reveal an otherwise masked sumoylation site. However, we did not observe any sumoylation of multiple mutant lamin C, strongly suggesting that lamin A/C is not sumoylated by sumo1.

The mutant intranuclear lamin A/C aggregates caused by the *in vitro* over-expression of two DCM associated *LMNA* mutants, p.Asp192Gly-DCM and p.Gln353Lys, as well as the two EDMD-associated *LMNA* mutants, p.His222Pro and p.Arg386Lys, sequester the sumo1 protein. Over-expression of sumo1 with wild-type mutant lamin A and C showed normal localization of lamin A/C with no trapping of sumo1, as did expression of the p.Leu85Arg-DCM mutant. The p.His222Pro mutant lamin A/C partially localizes to the nuclear envelope and forms intranuclear aggregates that sequester sumo1. The intracellular aggregates of p.His222Pro mutant lamin A/C were not previously seen in either cardiac or skeletal muscle from the *Lmna* ^H222P/H222P^ mouse [20]. The over-expression of mutant lamins might allow the amplification of the changes in lamin filament formation. It has been recently shown that the lamin A/C relocation from nuclear foci to the nuclear rim during embryonic development was blocked in the *Lmna* ^ΔK32/ΔK32^ muscular dystrophy mouse model, maintaining cells in an immature state [26]. Indeed in *Lmna* ^H222P/H222P^ primary myoblasts, we also observed an abnormal nuclear rim and/or intranuclear aggregation of both endogenous and exogenously expressed sumo1 as was seen in C2C12 cells. In skeletal muscle cross-sections, we observed a non homogeneous localization of sumo1 in approximately 30% of cells with intranuclear and nuclear envelope aggregates. In human tissues of laminopathic patients, the percentage of abnormal nuclei ranges from 0% to approximately 30% [1]. Certain sumoylation-desumoylation enzymes localize to the NPCs [27] and the proper assembly and positioning of NPCs is dependent on lamin A/C [28]. In fact, ubc9, which is critical for conjugation of sumo1 to targets as well for substrate specificity, is found both within the nucleus and at NPCs [27]. Furthermore, we have shown in C2C12 cells that ubc9 co-localizes with wild-type and mutant lamin A/C. SENP1 and SENP2 are sumo-specific proteases that de-sumoylate modified proteins and are found in the nucleus and at the nucleoplasmic face of the NPCs, respectively [29], [30]. Also, RanBP2, a protein with sumo E3 ligase function also localizes to the cytosolic filaments of the NPCs [31]. Therefore, mutant lamin A/C may be altering the assembly or function of the lamina, ubc9, and NPCs and thus disrupting the location of proteins or protein-protein interactions of the enzymes involved in the sumoylation cascade. This hypothesis is currently supported by the intermittent occurrence of ruptures in the nuclear envelope in dermal fibroblast cultures of patients carrying selected *LMNA* mutations [32]. These ruptures are accompanied by the loss of cellular compartmentalization [32].

The sequestration of sumo1 within mutant lamin aggregates paralleled with an increase in the steady-state levels of sumoylated proteins in nuclear extracts of C2C12 cells. There was also a modest increase in the amount of non-conjugated sumo1. Our results suggest that the trapping of sumo1 in the lamin A/C aggregates may conceal sumoylated proteins from normal de-conjugation and/or sumo1 degradation. Furthermore, although not sequestered within the aggregates, we found ubc9 to co-localize with both wild-type and mutant lamin A/C regardless of aggregation phenotype. This co-localization of ubc9 at lamin aggregates may be maintaining or promoting the higher levels of sumoylation as observed. Indeed, global levels of sumo modification can be altered by affecting ubc9 activity [33]. As there are hundreds of proteins known to undergo sumoylation, the consequences of sumo1 mislocalization could have disastrous consequences on the regulation of many cellular processes. Previous research in cardiac and skeletal muscles of the *Lmna* ^H222P/H222P^ mouse demonstrated an increase in the nuclear accumulation of Smad proteins, which are potent effectors of the TGFβ_1_ signalling cascade correlating with increased fibrosis in the mice [20], [34]. Both the TGFβ receptor type I (TβRI), which activates the Smad proteins, as well as Smad4 are sumoylated [35], [36]. TβRI is sumoylated in response to TGFβ and amplifies the signal by modulating gene expression [36]. However, there are conflicting reports as to whether sumoylation stimulates or represses Smad4 [35], [37], [38], [39], [40]. The nuclear accumulation of Smad proteins may be the result of altered Smad4 and/or TβRI sumoylation in the presence of lamin A/C mutants.

The cellular dynamics behind the development of striated-muscle specific laminopathies is not well understood. Our results provide further insight into the tissue-specific cellular regulation that is altered as a result of the *LMNA* mutations, suggesting that disease-associated mutations in the *LMNA* gene mediate a mislocalization of sumo1, ubc9, and likely sumoylated proteins in a mutation-dependent manner. Consequently, deficient deconjugation and/or degradation of sumo1 occurs, indicating a misregulation of the sumoylation process. As sumoylation is a highly conserved and tightly regulated cellular process with numerous targets, we propose that altered sumo1 dynamics may play a role in the pathophysiology of laminopathies.

## Materials and Methods

### Ethics Statement

The animal ethics approval form’s authorization # 75-636 was delivered to Dr. Gisele Bonne by the Préfet de Police’s office and by order of the Directeur Départemental des Services Vétérinaires de Paris. It is valid until April 17^th^, 2013. As we previously reported [20], all experiments were performed in accordance with the guide for the care and use of laboratory animals published by the NIH (publication No. 85-23, revised 1985).

### Expression Vector Preparation

Full length lamin A, lamin C, and sumo1 cDNA was previously cloned into fluorescent expression vectors (pECFP-C1, pDsRed2-C1, and pEYFP-N1 from Clonetech laboratories) as described previously [12]. The p.Leu85Arg, p.Asp192Gly, and p.Arg386Lys point mutations were previously introduced into the lamin A and C cDNA by site-directed mutagenesis as described previously [12]. The ubc9-HA vector was a kind gift from Peter Howley as seen in Yasugi and Howley (1996) [41]. The p.His222Pro point mutation was introduced into the lamin A and C cDNA by site-directed mutagenesis (Stratagene) with forward primer 5′-TCTCCACCAGTCGGGTCTCAGGACGGCGCTTGGTCTCACGC-3′ and reverse primer 5′-GCGTGAGACCAAGCGCCGTCCTGAGACCCGACTGGTGGAGA-3′. The p.Gln353Lys point mutation was introduced using the forward primer 5′-CCGAGATGCGGGCAAGGATGAAGCAGCAGCTGGACGAGTAC-3′ and reverse primer 5′-GTACTCGTCCAGCTGCTGCTTCATCCTTGCCCGCATCTCGG-3′. All the inserts were systematically verified by sequencing.

### Cell Culture and Transfection

C2C12 mouse myoblasts and COS7 monkey kidney cells (ATCC# CRL-1772 and CRL-1651) were cultured in Dulbecco’s Modified Eagle Medium (DMEM) and transiently transfected using Metafectene Pro (Biontex). Primary mouse myoblasts were harvested from wild-type and *Lmna* ^H222P/H222P^ mutant mice as described previously [22], cultured in Myo-1 medium, and transfected using electroporation (Amaxa). Cells were harvested 24 hours post-transfection and either nuclear protein (Active Motif) or total protein was extracted. N-ethylmaleimide (NEM) was added to certain extractions to preserve sumoylation [42].

### Co-immunoprecipitation

500 µg nuclear proteins were incubated with 25 µl washed Protein G magnetic beads (Dynal - Invitrogen) for 1 hour at 4°C to reduce nonspecific binding. Each sample was incubated rotating at room temperature for three hours with 2 µg primary antibody (anti-GFP sc-9996, anti-lamin A/C sc-6215, Santa Cruz). Then, the protein/antibody solution was incubated with 25 µl washed Protein G magnetic beads for 1 hour at room temperature. Samples were washed three times in 1X PBS buffer supplemented with 50 mM NaCl and 0.1% Tween, boiled with SDS sample buffer with DTT at 95°C for five minutes, cooled, and separated by magnet prior to immunoblotting.

### Immunoblotting

Primary antibodies used included goat anti-lamin A/C, rabbit anti-SP3, mouse anti-sumo1, mouse anti-GFP (Santa Cruz Biotechnology 6215, 644, 5308, and 69779), mouse anti-GapDH (ABM G041), and mouse anti-emerin (Novocastra NCL-EMERIN). Secondary HRP-conjugated antibodies used included goat anti-mouse, rabbit anti-goat, or goat anti rabbit (Santa Cruz 2005, 2768, 2004). Visualization was performed using ECL detection reagent (Amersham) in the Fluorochem chemiluminescent imager (Alpha Innotech). Blots were analyzed for densitometry using the AlphaEase program. GapDH was used as protein loading controls.

### Densitometry and Statistical Analysis

Samples were normalized to GapDH housekeeping protein to account for protein loading differences. As densitometry values vary greatly between blots, wild-type values were set at 100% and mutant values were compared against the wild-type as a ratio. As these calculations have not been previously shown to follow a normal distribution, a non-parametric Wilcoxon rank test was used with statistical significance set at p<0.01.

### Immunostaining and Fluorescent Microscopy

C2C12 images were captured on an Olympus Fluoview FV1000 confocal microscope using the Olympus FV-10 acquisition software. To avoid visual-based interpretation, quantitative colocalization analysis of lamin A and C and sumo1 was performed using the ImageJ- 1.46r software by at least two independent investigators. Briefly, images were opened and changed from 16-bit to 8-bit. Then the nucleus of interest was selected and the outside cleared (command “clear outside”) in order to get rid of extraneous objects near an item of interest. The background was then subtracted (command “subtract background”). The colocalization analysis was made using plugin “colocalization threshold” which allows calculation of the overlapping pixels intensity between the two channels, channel 1 (Ch1) and channel 2 (Ch2), and the Mander’s coefficient using thresholds of the two channels (tM1 and tM2). Mander’s coefficients indicate an overlap of the signals which represents the degree of colocalization. In every experiment, the green fluorescence emitted by sumo1 was primarily detected in Ch1 and the red fluorescence emitted by the lamins A and C was primarily detected in Ch2.

Cryostat soleus muscle cross sections 6 µm thick were obtained from *Lmna* ^+/+^ and *Lmna* ^H222P/H222P^ mice [20]. Prior to staining, slides were treated with 0.01 M citric acid (pH 6.0) and incubated with 0.05 mg/ml mouse Fab. Tissue sections and primary mouse myoblasts were incubated with 1∶50 mouse anti-SUMO1 (Santa Cruz Biotechnology 6215) primary antibody overnight at 4°C and then 1∶200 Alexa Fluor 568 goat anti-mouse (Invitrogen A-21124) secondary antibody for 30–60 min in the dark. Images were captured on a CarlZeiss Axiophot2 or Z1 Imager fluorescent microscopes.
